# HIV Care and Viral Suppression During the Last Year of Life: A Comparison of HIV-Infected Persons Who Died of HIV-Attributable Causes With Persons Who Died of Other Causes in 2012 in 13 US Jurisdictions

**DOI:** 10.2196/publichealth.6206

**Published:** 2017-01-24

**Authors:** William K Adih, H Irene Hall, Richard M Selik, Xiuchan Guo

**Affiliations:** ^1^ Division of HIV/AIDS Prevention National Center for HIV/AIDS, Viral Hepatitis, STD, and TB Prevention Centers for Disease Control and Prevention Atlanta, GA United States

**Keywords:** HIV, AIDS, causes of death, care

## Abstract

**Background:**

Little information is available about care before death among human immunodeficiency virus (HIV)–infected persons who die of HIV infection, compared with those who die of other causes.

**Objective:**

The objective of our study was to compare HIV care and outcome before death among persons with HIV who died of HIV-attributable versus other causes.

**Methods:**

We used National HIV Surveillance System data on CD4 T-lymphocyte counts and viral loads within 12 months before death in 2012, as well as on underlying cause of death. Deaths were classified as “HIV-attributable” if the reported underlying cause was HIV infection, an AIDS-defining disease, or immunodeficiency and as attributable to “other causes” if the cause was anything else. Persons were classified as “in continuous care” if they had ≥2 CD4 or viral load test results ≥3 months apart in those 12 months and as having “viral suppression” if their last viral load was <200 copies/mL.

**Results:**

Among persons dying of HIV-attributable or other causes, respectively, 65.28% (2104/3223) and 30.88% (1041/3371) met AIDS criteria within 12 months before death, and 33.76% (1088/3223) and 50.96% (1718/3371) had viral suppression. The percentage of persons who received ≥2 tests ≥3 months apart did not differ by cause of death. Prevalence of viral suppression for persons who ever had AIDS was lower among those who died of HIV but did not differ by cause for those who never had AIDS.

**Conclusions:**

The lower prevalence of viral suppression among persons who died of HIV than among those who died of other causes implies a need to improve viral suppression strategies to reduce mortality due to HIV infection.

## Introduction

As human immunodeficiency virus (HIV)–infected persons are surviving to older ages, the spectrum of causes of death among them is changing—the proportion of deaths in which HIV infection was the underlying cause has decreased, while the proportion of deaths due to causes not clearly attributable to HIV has increased [1-3]. Retention in medical care and effective treatment to achieve a suppressed viral load are essential to reduce morbidity and mortality, as well as the potential for onward transmission of the virus [4]. However, little information is available about care and outcomes before death among persons living with HIV who eventually died of either HIV-attributable or other causes. Assessment of predeath care by cause of death can inform opportunities for intervention. This study is an update on a recent study that examined HIV care within the year before death [5]. We expanded the investigation to compare HIV-infected persons who died of an HIV-attributable underlying cause with those who died of another underlying cause with respect to (1) disease stage within 12 months before death and (2) measures of care in terms of frequency of CD4 T-lymphocyte counts or viral load measurements and viral suppression.

## Methods

All US states and the District of Columbia require reporting of cases of HIV infection to their health departments; however, not all have mandatory reporting of all values of CD4 T-lymphocyte cell counts and viral load test results by laboratories. We used data reported to the National HIV Surveillance System (NHSS) of the Centers for Disease Control and Prevention through July 2015 from 13 jurisdictions with mandated laboratory reporting of all results from HIV-related tests to their HIV surveillance programs and that also collected cause of death information (California, District of Columbia, Hawaii, Iowa, Louisiana, Maryland, Michigan, Missouri, New Hampshire, New York, South Carolina, Texas, West Virginia). The analysis was restricted to persons who died in 2012, were ≥13 years old at the time of death, and resided in the selected areas at both diagnosis and death. The purpose of the residential restriction was to enhance the completeness of data on laboratory test results, because laboratories report test results to the health departments of the jurisdictions corresponding to the patient’s residential address reported by the health care provider. Among the health departments of these 13 jurisdictions, at least 85% of the deaths they reported to NHSS had data on the underlying cause, which they obtained by linking HIV surveillance data with either state or local vital records data or the National Death Index. The health departments reported the data to NHSS without key personal identifiers (eg, name, Social Security number) that they used to link HIV cases to death records and laboratory test results. Causes of death were identified by codes in the *International Classification of Diseases, Tenth Revision (ICD-10)* [6]. We classified a death as “HIV-attributable” if the reported underlying cause was HIV infection, indicated either explicitly (by an ICD code for HIV infection itself) or implicitly (by an ICD code for an AIDS-defining opportunistic illness or immunodeficiency—cell-mediated or unspecified type, not an antibody-mediated or congenital type). We assumed HIV infection was underlying an opportunistic illness or immunodeficiency because all the decedents had HIV infection reported to NHSS, even if HIV was not mentioned on the death certificate. If the underlying cause was known but was not HIV infection, we classified the death as “non–HIV-attributable death” (and the underlying cause as “other” than HIV infection; Multimedia Appendix 1). Persons missing information on underlying causes of death were excluded from the analyses. Stage of disease was based on the most recent CD4 cell count or percentage, and was defined in a reversible way, so that a person whose HIV disease had previously met the criteria for stage 3 (AIDS) could be reclassified in stage 1 if the most recent CD4 cell count was ≥500 cells/µL [7]. Persons were considered to be “in care” within 12 months before death if they had ≥1 CD4 or viral load test result in that period and “in continuous care” if they had ≥2 CD4 or viral load test results at least 3 months apart within the last 12 months before death [8]. Viral suppression (defined as <200 copies/mL) was based on the most recent viral load in the 12 months before death.

We assessed indicators overall and by sex, age, race/ethnicity, and transmission category (male-to-male sexual contact, ie, men who had sex with men or MSM; injection drug use; MSM and injection drug use; heterosexual contact; and other). We also determined care and viral suppression by disease severity (whether a person’s infection had ever been classified as stage 3 disease, AIDS), the length of time since diagnosis of HIV infection, and urban versus rural area of residence at the time of diagnosis (metropolitan statistical area population ≥500,000; metropolitan statistical area population 50,000-499,999; and nonmetropolitan population <50,000). Using log-binomial regression, we calculated prevalence ratios and 95% CIs to determine statistical differences on measures of care between persons who died of HIV-attributable underlying causes and those who died of non–HIV-attributable underlying causes. To control for potential confounding covariates, the analyses were adjusted for sex, age at death, race/ethnicity, transmission category, and ever AIDS. Analyses were adjusted for missing risk factor information. Analyses were performed using SAS version 9.3 statistical software (SAS Institute Inc), with the GenMod procedure for the log-binomial regression.

## Results

Among 6594 persons who died in 2012 with diagnosed HIV infection, 48.88% (3223/6594) died of HIV-attributable underlying causes and 51.12% (3371/6594) died of non–HIV-attributable underlying causes. Of those who died of HIV-attributable causes, most were male (2374/3223, 73.66%) and ≥40 years old at death (2673/3223, 82.94%); their racial/ethnic distribution was 47.35 % (1526/3223) non-Hispanic black or African American, 23.30% (751/3223) non-Hispanic white, 22.25% (717/3223) Hispanic or Latino, and 7.11% (229/3223) other; 40.80% (1315/3223) were MSM (Table 1). Among those who died of non–HIV-attributable causes, most were male (2493/3371, 73.95%) and ≥40 years old at death (3064/3371, 90.89%); their racial/ethnic distribution was 45.57% (1536/3371) non-Hispanic black or African American, 29.01% (978/3371) non-Hispanic white, 18.30% (617/3371) Hispanic or Latino, and 7.12% (240/3371) other; 36.78% (1240/3371) were MSM (Table 2). Overall, the percentage of persons with late-stage disease (stage 3, AIDS, based on the most recent indicator, ie, CD4 test or opportunistic illness diagnosis) in the 12 months before death was more than twice as great among persons who died of HIV-attributable causes (65.28%, 2104/3223; Table 1) as among persons who died of non–HIV-attributable causes (30.88%, 1041/3371; Table 2). The percentage with stage 3 disease was similarly higher in almost all demographic groups and transmission categories of persons who died of HIV-attributable causes than in their counterparts who died of other causes.

**Table 1:** Most recent stage of disease within 12 months before death, among persons aged ≥13 years who died of HIV-attributable causes in 2012, in 13 US jurisdictions.

Decedent characteristics		Most recent stage^a^ of disease before HIV-attributable death^b^									
		Total		Stage 1 (CD4^c^≥500 cells/µL or ≥29%)		Stage 2 (CD4 200-499 cells/µL or 14%-28%)		Stage 3 (AIDS; OI^d^ or CD4<200 cells/µL or <14%)		Unknown	
		n	%^e^	n	%^f^	n	%^f^	n	%^f^	n	%^f^
Total		3223	100	244	7.6	542	16.8	2104	65.3	333	10.3
**Sex**											
	Male	2374	73.7	175	7.4	394	16.6	1543	65.0	262	11.0
	Female	849	26.3	69	8.1	148	17.4	561	66.1	71	8.4
**Age (years) at death, year-end 2012**											
	13-29	154	4.8	7	4.5	18	11.7	121	78.6	8	5.2
	30-39	396	12.3	8	2.0	40	10.1	321	81.1	27	6.8
	40-49	914	28.4	58	6.3	112	12.3	666	72.9	78	8.5
	50-59	1107	34.3	90	8.1	201	18.2	681	61.5	135	12.2
	≥60	652	20.2	81	12.4	171	26.2	315	48.3	85	13.0
**Race or ethnicity**											
	American Indian or Alaska Native	2	0.1	0	0	0	0	2	100	0	0
	Asian	20	0.6	0	0	1	5.0	17	85.0	2	10.0
	Black or African American	1526	47.3	112	7.3	239	15.7	1030	67.5	145	9.5
	Hispanic or Latino^g^	717	22.2	43	6	124	17.3	483	67.4	67	9.3
	Native Hawaiian or other Pacific Islander	3	0.1	0	0	0	0	2	66.7	1	33.3
	White	751	23.3	74	9.9	143	19.0	432	57.5	102	13.6
	Multiple races	204	6.3	15	7.4	35	17.2	138	67.6	16	7.8
**Transmission category^h^**											
	Male-to-male sexual contact (MSM^i^)	1315	40.8	98	7.4	216	16.4	854	65.0	148	11.2
	Male injection drug use	513	15.9	43	8.3	86	16.8	333	64.7	52.7	10.3
	Female injection drug use	305	9.5	26	8.5	63	20.5	189	61.8	28	9.2
	MSM^i^ and injection drug use	251	7.8	19	7.6	48	19.2	154	61.4	30	11.8
	Male heterosexual contact	271	8.4	12	4.4	42	15.4	188	69.3	30	10.9
	Female heterosexual contact	521	16.2	42	8.0	81	15.4	359	68.9	40	7.6
	Other	47	1.4	6	11.8	7	15.3	28	60.9	6	12.0
**Ever AIDS**											
	Yes	2946	91.4	171	5.8	448	15.2	2104	71.4	223	7.6
	No	277	8.6	73	26.4	94	33.9	0	0	110	39.7

^a^Stage of disease within 12 months before death based on most recent CD4 test performed.

^b^HIV-attributable deaths were those for which HIV infection, AIDS-indicative opportunistic illness, or immunodeficiency was the underlying cause.

^c^CD4: CD4^+^T-lymphocyte count (cells/µL) or percentage.

^d^OI: opportunistic illness (ie, AIDS-defining condition).

^e^Column percent.

^f^Row percent.

^g^Hispanic or Latino can be of any race.

^h^Data on transmission category statistically adjusted to account for missing transmission category.

^i^MSM: men who had sex with men.

**Table 2:** Most recent stage of disease within 12 months before death, among persons aged ≥13 years who died of non–HIV-attributable causes in 2012.

Decedent characteristics		Most recent stage^a^ of disease before non–HIV-attributable death^b^									
		Total		Stage 1 (CD4^c^≥500 cells/µL or ≥29%)		Stage 2 (CD4 200-499 cells/µL or 14%-28%)		Stage 3 (AIDS; OI^d^ or CD4<200 cells/µL or <14%)		Unknown	
		n	%^e^	n	%^f^	n	%^f^	n	%^f^	n	%^f^
Total		3371	100	686	20.4	987	29.3	1041	30.9	657	19.5
**Sex**											
	Male	2493	74.0	482	19.3	741	29.7	782	31.4	488	19.6
	Female	878	26.0	204	23.2	246	28.0	259	29.5	169	19.2
**Age (years) at death, year-end 2012**											
	13-29	74	2.2	16	21.6	17	23.0	20	27.0	21	28.4
	30-39	233	6.9	55	23.6	55	23.6	77	33.0	46	19.7
	40-49	786	23.3	163	20.7	208	26.5	264	33.6	151	19.2
	50-59	1292	38.3	259	20	393	30.4	416	32.2	224	17.3
	≥60	986	29.2	193	19.6	314	31.8	264	26.8	215	21.8
**Race or ethnicity**											
	American Indian or Alaska Native	2	0.1	0	0	0	0	1	50.0	1	50.0
	Asian	17	0.5	3	17.6	7	41.2	5	29.4	2	11.8
	Black or African American	1536	45.6	294	19.1	452	29.4	481	31.3	309	20.1
	Hispanic or Latino^g^	617	18.3	105	17.0	196	31.8	218	35.3	98	15.9
	Native Hawaiian or other Pacific Islander	3	0.1	1	33.3	0	0	2	66.7	0	0
	White	978	29	230	23.5	262	26.8	264	27.0	222	22.7
	Multiple races	218	6.5	53	24.3	70	32.1	70	32.1	25	11.5
**Transmission category^h^**											
	Male-to-male sexual contact (MSM^i^)	1240	36.8	263	21.2	371	29.9	373	30.1	234	18.8
	Male injection drug use	677	20.0	110	16.3	202	29.9	226	33.5	138	20.4
	Female injection drug use	397	11.8	85	21.3	121	30.5	112	28.2	79	20.0
	MSM^i^ and injection drug use	287	8.5	56	19.6	85	29.5	94	32.8	52	18.1
	Male heterosexual contact	273	8.1	52	19.1	76	27.8	85	31.1	60	22.0
	Female heterosexual contact	471	14.0	118	24.9	122	26.0	143	30.4	88	18.8
	Other	27	0.8	3	11.7	11	39.6	7	26.7	6	22
**Ever AIDS**											
	Yes	2564	76.1	401	15.6	779	30.4	1041	40.6	343	13.4
	No	807	23.9	285	35.3	208	25.8	0	0	314	38.9

^a^Stage of disease within 12 months before death based on most recent CD4 test performed.

^b^Non–HIV-attributable deaths were all deaths for which the underlying cause was known other than those for which the underlying cause was HIV disease, an AIDS-indicative opportunistic illness, or immunodeficiency.

^c^CD4: CD4^+^T-lymphocyte count (cells/µL) or percentage.

^d^OI: opportunistic illness (ie, AIDS-defining condition).

^e^Column percent.

^f^Row percent.

^g^Hispanic or Latino can be of any race.

^h^Data on transmission category statistically adjusted to account for missing transmission category.

^i^MSM: men who had sex with men.

Overall, 91.34% (2944/3223) and 82.74% (2789/3371) of persons who died of HIV-attributable or non–HIV-attributable causes, respectively, had care within 12 months before death (≥1 CD4 or viral load test; Table 3). The percentage of persons who received continuous care (≥2 CD4 or viral load tests, 3 months apart) was similar among persons who died of HIV-attributable and non–HIV-attributable causes (66.15%, 2132/3223 and 65.89%, 2221/3371, respectively; Table 4). The percentage of persons with viral suppression was substantially lower among those who died of HIV-attributable causes (33.76%, 1088/3223) than among those who died of non–HIV-attributable causes (50.96%, 1718/3371; adjusted prevalence ratio 0.69, 95% CI 0.65-0.73; Table 4). Prevalence of viral suppression for persons who ever had stage 3 (AIDS) was lower among those who died of HIV but did not differ by cause of death for persons who never had stage 3 (AIDS).

The percentages with ≥1 CD4 or viral load test was ≥74% for most demographic groups and transmission categories but somewhat lower for persons who never had stage 3 disease (AIDS; 69.0%, 191/277 and 64.7%, 522/807 among persons who died of HIV-attributable and non–HIV-attributable causes, respectively) and persons whose diagnosis of HIV infection was ≤12 months before their death due to non–HIV-attributable causes (67.8%, 124/183).The percentage of persons with viral suppression was lower in most demographic and behavioral groups of persons who died of HIV-attributable causes than in their counterparts of persons who died of other causes.

Most persons in this study had HIV infection diagnosed more than 5 years before death (2368/3223, 73.47% and 2762/3371, 81.93% among those who died of HIV-attributable and non–HIV-attributable causes, respectively; Table 3).

**Table 3:** Care and viral suppression within 12 months before death, among persons aged ≥13 years, comparing those who died of HIV-attributable causes with those who died of non–HIV-attributable causes in 2012, in 13 US jurisdictions: “in care” (≥1 CD4 or viral load test).

Decedent characteristics		Total		≥1 CD4 or VL^a^ test						Unknown if had CD4 or VL test			
		HIV deaths^b^	Non-HIV deaths^c^	HIV deaths		Non-HIV deaths		APR^d^	95% CI^e^	HIV deaths^b^		Non-HIV deaths^c^	
		n	n	n	%	n	%			n	%	n	%
Total		3223	3371	2944	91.3	2789	82.7	1.05	1.03-1.06	279	8.7	582	17.0
**Sex**													
	Male	2374	2493	2159	90.9	2060	82.6	1.05	1.03-1.07	215	9.1	433	17.4
	Female	849	878	785	92.5	729	83.0	1.05	1.02-1.09	64	7.5	149	17.0
**Age (years) at death, year-end 2012**													
	13-29	154	74	146	94.8	55	74.3	1.02	0.92-1.14	8	5.2	19	25.7
	30-39	396	233	371	93.7	193	82.8	1.02	0.97-1.07	25	6.3	40	17.2
	40-49	914	786	848	92.8	655	83.3	1.06	1.03-1.10	66	7.2	131	16.7
	50-59	1107	1292	999	90.2	1095	84.8	1.03	1.00-1.06	108	9.8	197	15.2
	≥60	652	986	580	89.0	791	80.2	1.09	1.05-1.13	72	11.0	195	19.8
**Race or ethnicity**													
	Black or African American	1526	1536	1397	91.5	1254	81.6	1.05	1.02-1.08	129	8.5	282	18.4
	Hispanic or Latino^f^	717	617	661	92.2	529	85.7	1.04	1.00-1.08	56	7.8	88	14.3
	White	751	978	671	89.3	790	80.8	1.07	1.03-1.11	80	10.7	188	19.2
	Other races	229	240	215	93.9	216	90.0	1.02	0.97-1.07	14	6.1	24	10.0
**Transmission category^g^**													
	Male-to-male sexual contact (MSM^h^)	1315	1240	1198	91.1	1041	84.0	1.03	1.01-1.06	117	8.9	199	16.0
	Male injection drug use	513	676	470	91.6	548	81.1	1.08	1.03-1.12	43	8.4	128	18.9
	Female injection drug use	305	397	281	92.0	327	82.2	1.08	1.02-1.14	24	7.9	70	17.6
	MSM^h^ and injection drug use	251	287	225	89.6	240	83.7	1.05	0.98-1.12	26	10.4	47	16.4
	Male heterosexual contact	271	273	246	90.6	215	79.1	1.06	1.00-1.12	25	9.2	58	21.2
	Female heterosexual contact	521	471	486	93.2	394	83.6	1.04	0.99-1.08	35	6.7	77	16.3
	Other	47	27	39	83.9	23	85.3	0.93	0.77-1.12	8	17.0	4	14.8
**Ever AIDS**													
	Yes	2946	2564	2753	93.4	2267	88.4	1.05	1.03-1.06	193	6.6	297	11.6
	No	277	807	191	69.0	522	64.7	1.05	0.96-1.16	86	31.0	285	35.3
**Time since HIV diagnosis**													
	≤12 months	450	183	410	91.1	124	67.8	1.34	1.21-1.49	40	8.9	59	32.2
	13-24 months	108	93	100	92.6	77	82.8	1.12	1.00-1.24	8	7.4	16	17.2
	3-5 years	297	333	281	94.6	251	75.4	1.26	1.17-1.34	16	5.4	82	24.6
	More than 5 years	2368	2762	2153	90.9	2337	84.6	1.07	1.04-1.09	215	9.1	425	15.4
**MSA^i^** **at diagnosis**													
	MSA (population ≥500,000)	2688	2937	2468	91.8	2445	83.2	1.05	1.03-1.07	220	8.2	492	16.8
	MSA (population 50,000-499,999)	397	337	352	88.7	268	79.5	1.07	1.01-1.13	45	11.3	69	20.5
	Nonmetropolitan area (population <50,000)	128	81	115	89.8	66	81.5	0.98	0.91-1.05	13	10.2	15	18.5
	Unknown	10	16	9	90.0	10	62.5	-		1	10.0	6	37.5

^a^VL: viral load (copies/mL).

^b^HIV deaths (HIV-attributable deaths) were those for which HIV infection, AIDS-indicative opportunistic illness, or immunodeficiency was the underlying cause of death.

^c^Non-HIV deaths (non–HIV-attributable deaths) were all other deaths for which the underlying cause was known.

^d^APR: adjusted prevalence ratio, adjusted for sex, age at death, race/ethnicity, transmission category, and ever AIDS.

^e^CI: confidence interval

^f^Hispanic or Latino can be of any race.

^g^Data on transmission category have been statistically adjusted to account for missing transmission category.

^h^MSM: Men who had sex with men.

^i^MSA: metropolitan statistical area.

**Table 4:** Care and viral suppression within 12 months before death, among persons aged ≥13 years, comparing those who died of HIV-attributable causes with those who died of non–HIV-attributable causes in 2012, in 13 US jurisdictions: “in continuous care” (≥2 CD4 or viral load tests at least 3 months apart).

Decedent characteristics		≥2 CD4 or VL^a^ test at least 3 months apart						VL <200 copies/mL					
		HIV deaths^b,j^		Non-HIV deaths^c,j^		APR^d^	95% CI^e^	HIV deaths^b,j^		Non-HIV deaths^c,j^		APR^d^	95% CI^e^
		n	%	n	%			n	%	n	%		
Total		2132	66.1	2221	65.9	0.96	0.92-0.99	1088	33.8	1718	51.0	0.69	0.65-0.73
**Sex**													
	Male	1532	64.5	1642	65.9	0.93	0.89-0.97	843	35.5	1324	53.1	0.69	0.64-0.74
	Female	600	70.7	579	65.9	0.99	0.93-1.06	245	28.9	394	44.9	0.69	0.61-0.79
**Age (years) at death, year-end 2012**													
	13-29	97	63.0	41	55.4	0.94	0.72-1.23	34	22.1	20	27.0	1.12	0.60-2.09
	30-39	248	62.6	129	55.4	0.99	0.86-1.15	69	17.4	98	42.1	0.42	0.32-0.56
	40-49	607	66.4	497	63.2	0.97	0.90-1.04	284	31.1	361	45.9	0.67	0.59-0.76
	50-59	750	67.8	888	68.7	0.95	0.90-1.00	396	35.8	672	52.0	0.66	0.60-0.73
	≥60	430	66.0	666	67.5	0.95	0.88-1.01	305	46.8	567	57.5	0.8	0.73-0.89
**Race or ethnicity**													
	Black or African American	1055	69.1	987	64.3	1.01	0.96-1.06	453	29.7	678	44.1	0.71	0.64-0.78
	Hispanic or Latino^f^	464	64.7	459	74.4	0.89	0.83-0.96	245	34.2	353	57.2	0.63	0.56-0.72
	White	458	61.0	600	61.3	0.97	0.90-1.05	309	41.1	559	57.2	0.72	0.65-0.80
	Other races	155	67.7	175	72.9	0.9	0.80-1.00	81	35.4	128	53.3	0.7	0.57-0.87
**Transmission category^g^**													
	Male-to-male sexual contact (MSM^h^)	813	61.8	806	65.0	0.9	0.85-0.96	456	34.7	696	56.1	0.65	0.59-0.71
	Male injection drug use	362	70.6	459	68.0	1.01	0.94-1.08	202	39.4	349	51.6	0.78	0.69-0.89
	Female injection drug use	230	75.3	268	67.5	1.05	0.96-1.16	94	30.7	181	45.5	0.72	0.59-0.88
	MSM^h^ and injection drug use	171	68.2	199	69.3	0.95	0.84-1.06	92	36.7	161	56.2	0.66	0.54-0.81
	Male heterosexual contact	171	63.3	167	61.2	0.95	0.84-1.08	82	30.3	109	40.0	0.79	0.62-1.00
	Female heterosexual contact	354	68.0	303	64.4	0.95	0.87-1.04	142	27.3	209	44.5	0.65	0.54-0.78
	Other	30	65.2	18	65.6	0.86	0.63-1.18	19	41.7	13	48.7	1.08	0.61-1.94
**Ever AIDS**													
	Yes	1986	67.4	1848	72.1	0.94	0.91-0.98	970	32.9	1395	54.4	0.66	0.62-0.71
	No	146	52.7	373	46.2	1.13	0.99-1.29	118	42.6	323	40.0	1.05	0.90-1.23
**Time since HIV diagnosis**													
	≤12 months	116	25.8	48	26.2	0.97	0.73-1.30	80	17.8	31	16.9	0.89	0.59-1.34
	13-24 months	75	69.4	66	71.0	0.99	0.80-1.22	39	36.1	46	49.5	0.68	0.47-0.98
	3-5 years	193	65.0	181	54.4	1	0.88-1.14	103	34.7	139	41.7	0.9	0.72-1.12
	More than 5 years	1748	73.8	1926	69.7	1.02	0.99-1.06	866	36.6	1502	54.4	0.71	0.67-0.76
**MSA^i^** **at diagnosis**													
	MSA (population ≥500,000)	1816	67.6	1977	67.3	0.96	0.93-1.00	941	35.0	1520	51.8	0.7	0.66-0.75
	MSA (population 50,000-499,999)	230	57.9	189	56.1	0.95	0.83-1.08	108	27.2	144	42.7	0.69	0.56-0.86
	Nonmetropolitan area (population <50,000)	79	61.7	47	58.0	0.9	0.72-1.11	36	28.1	48	59.3	0.43	0.31-0.60
	Unknown	7	70.0	8	50.0	-		3	30.0	6	37.5	-	

^a^VL: viral load (copies/mL).

^b^HIV deaths (HIV-attributable deaths) were those for which HIV infection, AIDS-indicative opportunistic illness, or immunodeficiency was the underlying cause of death.

^c^Non-HIV deaths (non–HIV-attributable deaths) were all other deaths for which the underlying cause was known.

^d^APR: adjusted prevalence ratio, adjusted for sex, age at death, race/ethnicity, transmission category, and ever AIDS.

^e^CI: confidence interval

^f^Hispanic or Latino can be of any race.

^g^Data on transmission category have been statistically adjusted to account for missing transmission category.

^h^MSM: men who had sex with men.

^i^MSA: metropolitan statistical area.

^j^Total HIV and non-HIV deaths (denominators) are shown in Table 3.

## Discussion

### Principal Findings

Our results indicated poorer outcome in terms of viral suppression in the last 12 months before death among persons who died of HIV-attributable causes, consistent with other studies [9]. Almost two-thirds (65.28%) of persons who died of HIV-attributable causes had late-stage disease (stage 3, AIDS) in the 12 months before death, compared with 30.88% of those who died of other causes, and the percentage with a suppressed viral load was lower among persons who died of HIV-attributable causes (33.76%) than among those who died of other causes (50.96%). This association between death due to HIV-attributable causes and both late-stage disease and lack of viral suppression could be explained by the latter two conditions being characteristic of late (delayed) diagnosis of HIV infection, inadequate care and treatment, inadequate adherence to medication regimens, or treatment failure [9,10].

Although a high percentage of persons who died received care, a low percentage of them had viral suppression, particularly among persons who died of HIV-attributable causes. This is consistent with other studies [9,10]. Although receipt of care might be expected to lead to viral suppression, the causal relationship may actually be in the reverse direction—being “in care” or “in continuous care,” as measured by frequency of CD4 or viral load tests, could be a marker for clinical deterioration with high viral loads, due to lack of adherence or treatment failure, which then resulted in more frequent care.

### Limitations

Our analyses were subject to some limitations. First, our analyses were based on data from 13 jurisdictions, representing 41% of all persons 13 years and older who died in 2012 in the United States, and, therefore, may not be representative of all persons with HIV who died in the United States. Second, cause of death information from death certificates may underestimate deaths due to HIV [11,12]. The finding that a substantial percentage (30.88%) of the persons who died of non–HIV-attributable causes had stage 3 (AIDS) suggests that some of these deaths might actually have been HIV-attributable. Conversely, HIV infection may have been characterized as underlying cause of death for some patients without actually playing a role in their death if the physicians who certified the deaths ignored the instructions on the death certificate to list as causes only those conditions that “resulted in” or “contributed to” death, or incorrectly assumed that HIV infection did so. Third, information was not available on highly active antiretroviral therapy (HAART) or treatment adherence. Fourth, CD4 and viral load testing may not adequately capture the full spectrum of HIV care in the last 12 months before death [5].

### Conclusions

HAART has prolonged the survival of HIV-infected persons by reducing deaths due to HIV-attributable causes [13]. To further decrease mortality, HIV-infected persons should seek early testing and, when diagnosed, be linked to care as soon as possible and be retained in such care so as to reduce the risk of death due to HIV and enable persons with HIV to have a life expectancy similar to that of persons without HIV [13]. This is in consonance with the recommendation by the Panel on Antiretroviral Guidelines for Adults and Adolescents that diagnosis of HIV be made early in the course of infection so as to initiate therapy early and at any CD4 count [14,15].

## Appendix

Multimedia Appendix 1

## Abbreviations

HAART: highly active antiretroviral therapy
HIV: human immunodeficiency virus
ICD-10: International Classification of Diseases, Tenth Revision
MSM: men who had sex with men
NHSS: National HIV Surveillance System
