# Supplementary material for: HIV Care and Viral Suppression During the Last Year of Life: A Comparison of HIV-Infected Persons Who Died of HIV-Attributable Causes With Persons Who Died of Other Causes in 2012 in 13 US Jurisdictions
Source: JMIR Public Health Surveill. 2017 Jan 24;3(1):e3. doi: 10.2196/publichealth.6206 (PMC5296618; doi:10.2196/publichealth.6206)
Supplement: Multimedia Appendix 1 [file publichealth_v3i1e3_app1.pdf]

Appendix. ICD-10 codes for underlying causes of deaths in persons diagnosed with HIV infection

Underlying cause of death	ICD-10 codes	No.	%
**HIV-attributable cause causes of death (Total)**		**3,223**	
I. Human immunodeficiency virus (HIV) disease	B20-B24, 098.7, R75	2,972	92.2
II. AIDS-indicative opportunistic illnesses (AIDS OI)			
Various types of pneumonia	J12-J18	85	2.6
Non-Hodgkin lymphoma, excluding follicular lymphoma	C83, C85	63	2.0
Dementia/Encephalopathy	F03, A86, G04.9, G93.4, G93.9	20	0.6
Cervical cancer	C53	18	0.6
Pneumocystosis	B59	13	0.4
Kaposi sarcoma	C46	8	0.2
Progressive multifocal leukoencephalopathy	A81.2	4	0.1
Wasting	R62, E46	4	0.1
Candidiasis	B37	3	0.1
Cytomegalovirus disease	B25	3	0.1
Cryptococcosis	B45	2	0.1
Salmonella infections	A02	1	<0.1
Histoplasmosis	B39	1	<0.1
III. Immunodeficiency	D84.9, D84.8	26	0.8
**Non-HIV-attributable causes of death (Total)**		**3371**	
Non-AIDS cancer	C00-C97 (except C46, C83, C85)	847	25.1
Heart disease	I11, I13, I20-I51 (except I46)	716	21.2
Unintentional injury or poisoning (Accidents)	V01-V99, W00-W99, X01-X59, Y85, Y86	420	12.5
Chronic liver disease	K70-K74	117	3.5
Self-harm (Suicide)	U03, Y87.0, X60-X84	101	3.0
Stroke	I60-I69	94	2.8
Assault (homicide)	U01,U02, X85-X99, Y00-Y09,Y87.1	91	2.7
Diabetes	E10-E14	86	2.6
Kidney disease	N00-N07, N17-N19, N25-N27	74	2.0
Other causes		825	24.5
